# Medium-throughput zebrafish optogenetic platform identifies deficits in subsequent neural activity following brief early exposure to cannabidiol and Δ^9^-tetrahydrocannabinol

**DOI:** 10.1038/s41598-021-90902-3

**Published:** 2021-06-01

**Authors:** Richard Kanyo, Md Ruhul Amin, Laszlo F. Locskai, Danika D. Bouvier, Alexandria M. Olthuis, W. Ted Allison, Declan W. Ali

**Affiliations:** 1 Centre for Prions and Protein Folding Disease, University of Alberta, Edmonton, AB T6G 2M8 Canada; 2 Department of Biological Sciences, University of Alberta, Edmonton, AB T6G 2E9 Canada; 3 Department of Medical Genetics, University of Alberta, Edmonton, AB T6G 2H7 Canada; 4 Neuroscience and Mental Health Institute, 4-120 Katz Group Centre, Edmonton, T6G 2E1 Canada

**Keywords:** Biological models, High-throughput screening, Imaging, Developmental biology, Risk factors, Development of the nervous system, Motor control

## Abstract

In light of legislative changes and the widespread use of cannabis as a recreational and medicinal drug, delayed effects of cannabis upon brief exposure during embryonic development are of high interest as early pregnancies often go undetected. Here, zebrafish embryos were exposed to cannabidiol (CBD) and Δ^9^-tetrahydrocannabinol (THC) until the end of gastrulation (1–10 h post-fertilization) and analyzed later in development (4–5 days post-fertilization). In order to measure neural activity, we implemented Calcium-Modulated Photoactivatable Ratiometric Integrator (CaMPARI) and optimized the protocol for a 96-well format complemented by locomotor analysis. Our results revealed that neural activity was decreased by CBD more than THC. At higher doses, both cannabinoids could dramatically reduce neural activity and locomotor activity. Interestingly, the decrease was more pronounced when CBD and THC were combined. At the receptor level, CBD-mediated reduction of locomotor activity was partially prevented using cannabinoid type 1 and 2 receptor inhibitors. Overall, we report that CBD toxicity occurs via two cannabinoid receptors and is synergistically enhanced by THC exposure to negatively impact neural activity late in larval development. Future studies are warranted to reveal other cannabinoids and their receptors to understand the implications of cannabis consumption on fetal development.

## Introduction

Cannabis is often portrayed as harmless, yet its health implications are not fully understood^1^. The extracts of the *Cannabis sativa* plant have been used for medical purposes for almost 5000 years, especially for the treatment of pain^2^. Despite controversy and incomplete knowledge of toxicities, cannabis has been legalized for recreational use in various jurisdictions. Cannabis is one of the most used illicit drugs during pregnancy, with an increase in consumption over recent years. Importantly, key phytocannabinoids such as Δ^9^-tetrahydrocannabinol (THC) and cannabidiol (CBD) can readily cross the placenta^3–8^. Moreover, CBD use is promoted due to its purported health benefits in various natural products including oils and food^9^.

The negative impacts of cannabis on embryonic development have been principally associated with the psychoactive compound THC^10^. Epidemiological and clinical studies associate maternal cannabis exposure with behavioural disturbances in the offspring and are linked to an increased risk of neuropsychiatric disorders^11^. Maternal exposure to THC in rats changes a series of behaviours in their offspring including water-induced grooming, increased light sensitivity, and altered exploratory behaviour^12^. Importantly, the negative impacts of cannabis may also occur via the non-psychotropic compound CBD, which disrupts motor-neuron development in zebrafish^13^. This latter study is of concern because it stands in contrast to reports that promote the positive health benefits of CBD, for example in the treatment of nausea during pregnancy^14,15^.

The mechanistic pathways by which THC and CBD are toxic during development remain elusive. Both of these cannabinoids have recently been linked to the sonic hedgehog signaling pathway in mice and zebrafish, and signal via the cannabinoid type 1 receptor (CB1R)^16^. THC binds to two distinct classes of G-protein coupled receptors, CB1R and CB2R, where it acts as a partial agonist^17^. Both receptor types are expressed in the nervous system, with CB1R being highly enriched in the CNS^18–20^. While CB2R is more commonly found in the peripheral nervous system^21^, it is also associated with the CNS^22–24^. CBD interacts with CB1R and CB2R, but with lower affinity relative to THC. CBD is also thought to antagonize cannabinoid-induced effects indirectly through other receptors^25–29^. Additional in vivo data on the signaling pathways of THC and CBD would highlight functionally important mechanisms.

Zebrafish embryos have several advantages that complement mammalian models in attempting to disentangle these issues. Experiments are relatively economical because many embryos are available. Exposure studies are simple as compounds can be easily added and removed from the incubation media that bathes the developing embryos. Further, embryos develop externally and experimental results are not confounded by maternal physiology or variable transport to the fetus. Translucent larvae provide opportunities to implement cutting-edge fluorescing calcium sensors and to measure neural activity. The endocannabinoid system plays a role in zebrafish development^30–32^. CB1R and CB2R have similar expression profiles in the CNS of zebrafish compared to mammals, and CB1R shares a 70% protein sequence identity with its human homolog^33^. Therefore, the zebrafish is a favorable model organism to further illuminate evolutionary conserved biological mechanisms.

Considering the anticipated effects of THC and CBD on developing embryos, we sought to develop high-throughput quantification of neural activity following early drug exposures. Here we deploy the calcium modulated photoactivatable ratiometric integrator (CaMPARI)^34^ to document the effects of THC and CBD on neural activity. CaMPARI is expressed exclusively in neuronal tissue due to a pan-neural promoter (*elavl3*, a.k.a. HuC) and is a direct read-out of relative neural activity. Green fluorescing CaMPARI undergoes photoconversion (PC) to a red fluorescing protein only when in the presence of high Ca^2+^ levels occurring coincidentally with user-controlled intense 405 nm light. This photoconversion is irreversible and thus creates a temporal snapshot of neural activity in freely swimming larvae. The CaMPARI photoconversion is subsequently quantified as a ratiometric red/green output, and this mitigates many technical challenges of imaging neural activity that are imposed by issues such as variable expression, tissue mounting, or tissue depth. These advantages combine to make CaMPARI uniquely adaptable for high-throughput quantifications. We optimized the workflow and photoconversion of CaMPARI to make it practical for high-throughput well-plate-formats. Our novel in vivo assay is scalable to measure neural activity via screens of compound libraries. The results were supplemented with a behavioral assay performed on the same larvae, which offers an independent proxy measure that is related to neural activity.

Here, we investigate the actions of THC and CBD on neural activity and test whether the in vivo effects of CBD are mediated via CB1R and CB2R in zebrafish. We report that early embryonic exposure to THC and CBD (until 10 h post-fertilization) impacts neural activity later in larval development (at 4 and 5 days post-fertilization (dpf)). The concentrations of THC and CBD used mimic plasma levels of human subjects with high cannabis consumption^35–37^, though complexity arises from estimating diffusion through multiple barriers (e.g. incubation media through the chorion and then into the plasma and tissues of the zebrafish embryo). We document the effects of THC and CBD when applied individually and in concert, to partially mimic the combination of cannabinoids in recreational cannabis. We probe the roles of CB1R and CB2R in this signaling using established receptor inhibitors, towards revealing their in vivo requirement amongst the various receptor mechanisms that have been identified from in vitro studies.

## Results

### High-throughput assessment of CaMPARI is a reliable metric of neural activity

**Figure 1:**
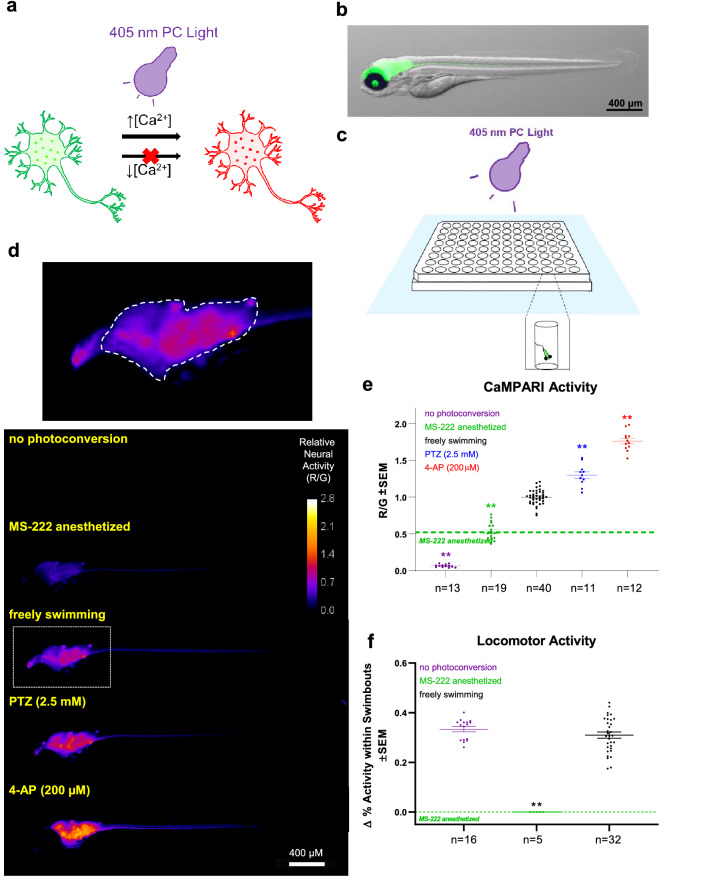
High-throughput quantification of neural activity in freely-swimming zebrafish larvae. (**a**) CaMPARI photoconverts from green to red fluorescing versions in the neuron only in the presence of both high intracellular calcium concentrations and a bright 405 nm light source. (**b**) Lateral view of green fluorescing CaMPARI merged with brightfield image shows exclusive expression in the CNS due to a pan-neural promoter (*elavl3*). (**c**) Larvae were transferred to 48 wells in the centre of a 96-well plate to ensure that the 405 nm LED Flood Array covers all larvae entirely. To minimize overheating, the plate was floating in a waterbath at 10 cm distant to the LED while CaMPARI was photoconverted (PC) by the LED Flood Array. (**d**) Lateral view of zebrafish with exemplar “CaMPARI Activity” heat maps. Heat maps show ratio of red/green (R/G) fluorescent output as indicated by the calibration bar and can be interpreted as relative neural activity. CNS regions with higher levels of neural activity translate to “hotter” pixels. Images were acquired using an automated INCell 2000 high-content microscope and reveal a reduction in neural activity when the larvae were anesthetized with MS-222 or an increase with convulsants, PTZ and 4-AP. Top, Enlarged lateral view from freely swimming larvae illustrating the optic tectum and hindbrain areas (dashed line) from which the R/G ratio was obtained. (**e**) Neural activity, inferred from the mean R/G ratio in optic tectum and hindbrain. CaMPARI activity is reduced to baseline in MS222-anaesthetized fish and photoconversion is undetectable when CaMPARI photoconverting light is omitted. Dashed green line represents mean for MS-222-anesthetized samples, which show a clear reduction in signal compared to freely swimming larvae and provides a baseline “near-zero” activity level for reference in subsequent experiments. Drivers of neural activity, PTZ and 4-AP induce a significant increase in CaMPARI activity. (**f**) Locomotor activity was measured using behavioural tracking software. PC light from the day prior at 4 days post-fertilization (dpf) did not affect locomotion at 5 dpf. MS-222 treatment abolished any swimbouts. Biological replicates are individual larvae = n. **Significantly different from freely swimming samples, *p* < 0.01.

An objective of this study was to gain insight into the effects of THC and CBD on neural activity during early embryonic development. We measured neural activity with CaMPARI, which photoconverts irreversibly from a green to a red fluorescing protein only if user-applied 405 nm light application coincides with high calcium levels (Fig. 1a)^34^. Red and green fluorescing CaMPARI are quantified as a ratio (referred here as “CaMPARI activity"). We expressed CaMPARI through a pan-neural promoter, *elavl3* exclusively in the CNS and thus it acts as a read-out of neural activity (Fig. 1b). Here, we optimized the use of CaMPARI zebrafish for an automated high-content INCell2000 plate-reader (Fig. 1c). We empirically determined that 4 dpf is a suitable age for quantifying CaMPARI, which provides an appropriate time for imaging (good CaMPARI expression and tissue clarity) coupled with a relatively late stage in CNS development.

CaMPARI activity was quantified and displayed as a heatmap consisting of the red/green ratio in fluorescing intensities. At 4 dpf, CaMPARI outputs in the optic tectum and hindbrain regions of larvae showed robust neural activity, whereas without photoconversion CaMPARI activity was nearly zero. To establish a baseline of near-zero neural activity, larvae were anesthetized with MS-222, and a significant reduction (*p* < 0.01) in red/green ratio was obtained compared to untreated freely swimming larvae. In contrast, drug-induced neural activity, with the established convulsants pentylenetetrazole (PTZ) or 4-aminopyridine (4-AP), showed a consistent increase in CaMPARI activity, even at concentrations considered to be minimal when inducing seizures (Fig. 1d, e)^38^.

We benchmarked our CaMPARI outputs against locomotor activity of zebrafish larvae, which is an established metric of neural activity^39^. We chose 5 dpf as an optimal developmental timepoint for analyzing locomotor activity, because younger larvae are largely inactive. Larvae were left in the same 96-well plate for assessing both CaMPARI and locomotion. Exposing larvae to photoconversion with LED light at 4 dpf had no impact on the mean locomotor activity at 5 dpf (Fig. 1f). Larvae anesthetized with MS-222 did not display any detectable swimbouts (Fig. 1f) and the level of neural activity was well correlated with locomotor activity (see below). Overall, these results show that CaMPARI optogenetic measures deployed in zebrafish larvae offer a reliable high-throughput tool for measuring neural activity.

### CBD and THC reduce subsequent neural activity

We assessed how embryonic exposure to cannabinoids impacts neural activity later in development. Here, the doses of CBD and THC aligned with our previous work^13^, reflecting high cannabis consumption in humans. Comparisons of our dosage to humans requires various considerations: (1) blood plasma concentrations of THC can peak up to 0.25 mg/l while smoking a single cigarette^35^; (2) the content of THC in cannabis has increased in the past 20 years; and (3) doses of intraperitoneally administered medical CBD can vary greatly, from 5 to 100 mg/kg, with a daily maximum of 1500 mg/kg^36,37^. The current study uses up to 6 µg/mL of THC and 3 µg/mL of CBD and absorption studies using Liquid Chromatography-Tandem Mass Spectrometry suggest that an estimated 0.1–10% of toxic compounds will pass through the chorion to reach the embryo^40,41^.

**Figure 2:**
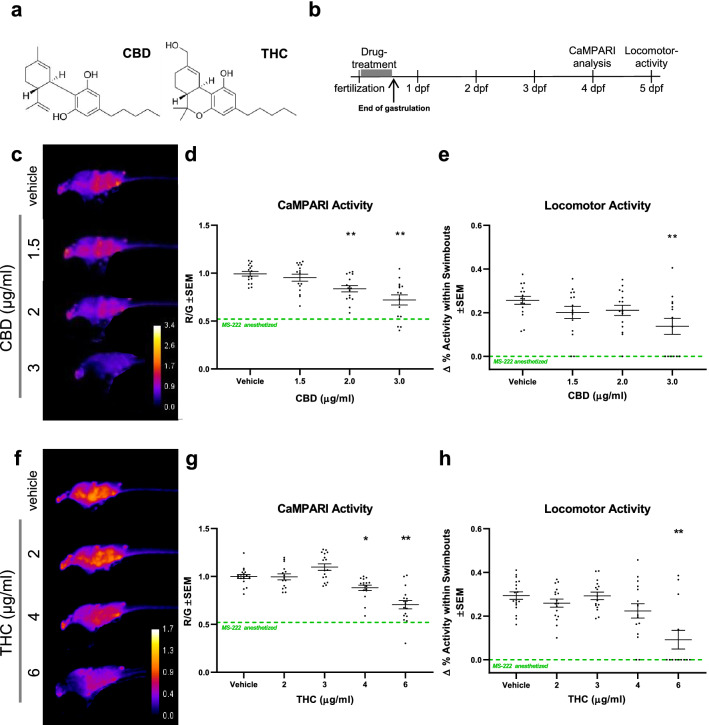
Early application of CBD and THC reduce subsequent neural activity and locomotor activity. (**a**) The chemical structures of key cannabinoids used in this study: CBD and THC. (**b**), Timeline of experimental set up where zebrafish embryos were treated with drugs between 0.5 and 1 h post-fertilization (hpf) and washed out at 10 hpf towards the end of gastrulation. CaMPARI imaging was obtained at 4 days post-fertilization (dpf) and locomotor activity was tracked at 5 dpf. (**c**) and (**f**), Representative CaMPARI activity maps obtained from treated larvae and corresponding quantification and statistics shown in (**d**) and (**g**), respectively. (**e**) and (**h**), Locomotor activity of the same larvae. (**c**) CBD reduces neural activity and locomotor activity as shown in (**d**) and (**e**). (**f**) THC requires a higher does than CBD to reduce neural activity and locomotor activity as shown in (**g**) and (**h**). CaMPARI activity heat maps show ratio of R/G channels as indicated by the calibration bar, with higher ratios (hotter colours) representing greater neural activity. Green-dashed lines depict mean baseline (zero) value for MS-222 anesthetized samples (From Fig. 1). Biological replicates are n = 8–19. *is *p* < 0.05; **p < 0.01 compared to the vehicle control (0.3% MeOH in experiments with CBD and 0.6% MeOH for THC).

To gain insight into the effects of CBD and THC separately (Fig. 2a), we applied a range of concentrations of each compound. This validation was necessary as CaMPARI has not been used previously to study the effects of cannabinoids. Compounds were added to the embryonic bathing media soon after egg fertilization at 0.5 hpf and were then washed out towards the end of gastrulation at 10 hpf. CaMPARI was imaged in 4 dpf larva and locomotor activity was assessed at 5 dpf (Fig. 2b). Animals that were exposed to CBD at concentrations of 2 µg/ml and 3 µg/ml exhibited a dose-dependent reduction in CaMPARI activity (Fig. 2c, d). These reductions in CaMPARI output are substantial when compared with the ~ 50% reduction we observed in anesthetized larvae, where little neural activity is expected. Coordinated with this, locomotion was also reduced starting at 1.5 µg/ml and was significant (*p* < 0.01) at 3 µg/ml (Fig. 2e). THC had a similar effect as CBD and also reduced neural activity at higher doses, 4 µg/ml and 6 µg/ml, (Fig. 2f, g). Locomotor activity exhibited a more extensive reduction at 6 µg/ml (Fig. 2h). We compared neural activity and locomotion in the same individuals exposed to effective doses of cannabinoids and found significant correlations (Fig. S2; CBD *r* = 0.52 (*p* < 0.01) and THC *r* = 0.71 (*p* < 0.01)). Indeed, most larvae that displayed reduced neural activity also showed reduced locomotion. Together, our findings show that both CBD and THC reduced neural activity when embryos were exposed early in development.

### Antagonistic effects of CBD on neural activity are enhanced when combined with sub-effective doses of THC

**Figure 3:**
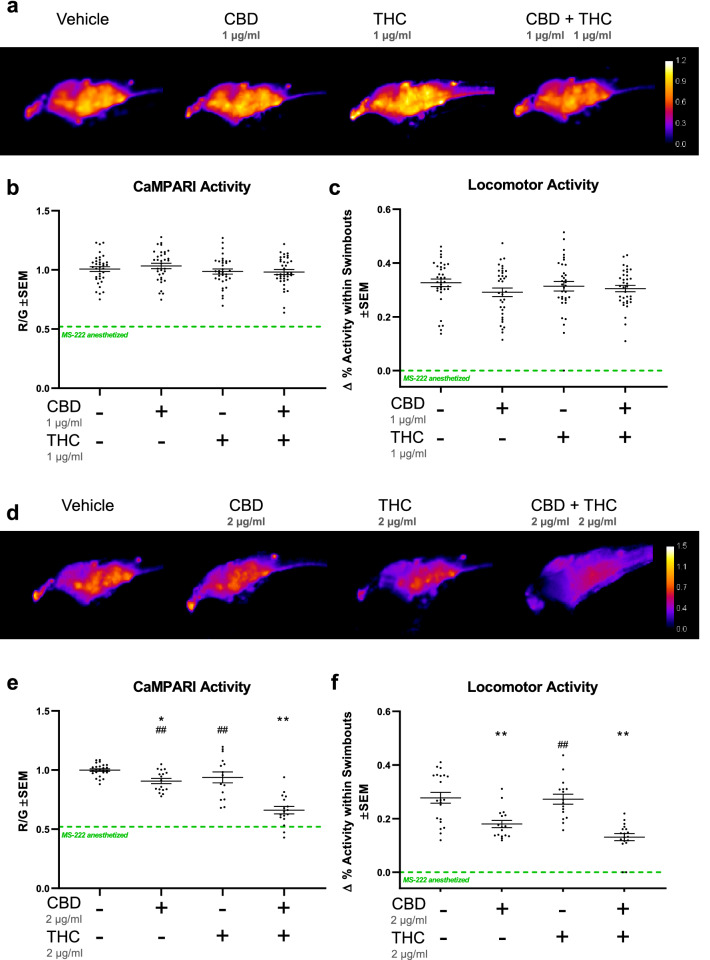
Antagonistic effect of CBD on neural activity is enhanced when combined with sub-effective doses of THC. Zebrafish larvae were exposed to a series of CBD and THC concentrations by themselves or in combination, which are mostly sub-effective, or in the case of CBD, were minimally effective. (**a**) and (**d**), Exemplar CaMPARI activity heat maps show an additive effect (minimizing neural activity) when exposed to 2 µg/ml (**d**) of each CBD and THC compared to CBD or THC by themselves (as plotted in (**e**)). When 1 µg/ml of 1:1 CBD and THC was applied, ratios show no additive effect as illustrated by quantifications and statistics in (**b**). (**c**) and (**f**), Locomotor activity from the same well at 5 dpf shows a clear reduction when CBD and THC is combined at 2 µg/ml each, but reduction mediated by CBD alone is almost as low as when combined with THC suggesting that CBD is the main component affecting locomotor activity. R/G is indicated by the calibration bar. Green-dashed lines depict mean values for MS-222-anesthetized samples (from Fig. 1). Biological replicates are n = 15–25. *compared to vehicle control (equal amount of MeOH in all experiments); ^#^ compared to CBD plus THC. One symbol is *p* < 0.05; Two symbols is *p* < 0.01.

Cannabis consumption during pregnancy exposes the fetus to CBD and THC in concert. In order to investigate whether CBD and THC have a combined effect that is different from either compound on its own, neural activity was measured when larvae were exposed to both cannabinoids simultaneously using the same timeline as the previous experiment (Fig. 2b). First, various sub-effective doses of CBD were applied from 0.5 to 1.5 µg/ml in the presence of 2 µg/ml THC (identified as sub-effective in Fig. 2). Preliminary dosage attempts revealed small, but significant differences with *p* < 0.01 and *p* < 0.05, respectively, on neural activity when combining 0.5 or 1.5 µg/ml CBD with 2.0 µg/ml THC (Fig. S3a, b). Locomotor activity also trended towards a dose-dependent reduction during concerted application of CBD and THC, which was significant at 1.5 µg/ml with *p* < 0.01 (Fig. S3c). Next, CBD and THC were added in a 1:1 ratio mixture at 1.0 and 2.0 µg/ml each. An enhanced antagonistic effect was revealed on neural activity when both CBD and THC were added at 2.0 µg/ml. CBD by itself at 2.0 µg/ml significantly (*p* < 0.05) reduced neural activity (Fig. 3d, e) consistent with results in Fig. 2d, but CBD and THC together, further reduced neural activity compared to CBD or THC alone (Fig. 3d, e; *p* < 0.01). The combined effects of CBD and THC seemed synergistic in that CBD by itself at 2.0 µg/ml, although significant, had a relatively small effect, and THC by itself at 2.0 µg/ml had no effect. However, when similar concentrations of CBD and THC were combined, neural activity was reduced to levels that were comparable with MS-222 anesthetized samples, suggesting an absence of neural activity. Locomotor activity was mainly affected by CBD with no additional reduction when compared to CBD and THC combined (Fig. 3f). No significant effect on neural activity or locomotor activity was obtained when 1.0 µg/ml of CBD and THC was applied (Fig. 3a–c).

Due to the effects on locomotion, we wanted to assess the integrity of neurons associated with locomotor networks. Reticulospinal neurons in the hindbrain were immunolabelled with the RMO44 antibody that targets NEFM, an established marker for neuronal damage^42,43^. Indeed, RMO44 immunolabelling was further reduced when CBD and THC were combined at 2.0 µg/ml suggesting that both CBD and THC affect neuronal health (Fig. S4). Together, these results suggest that CBD and THC are more potent in reducing neural activity when applied in combination.

### CB1R and CB2R are both required for CBD-induced reduction of locomotor activity

**Figure 4:**
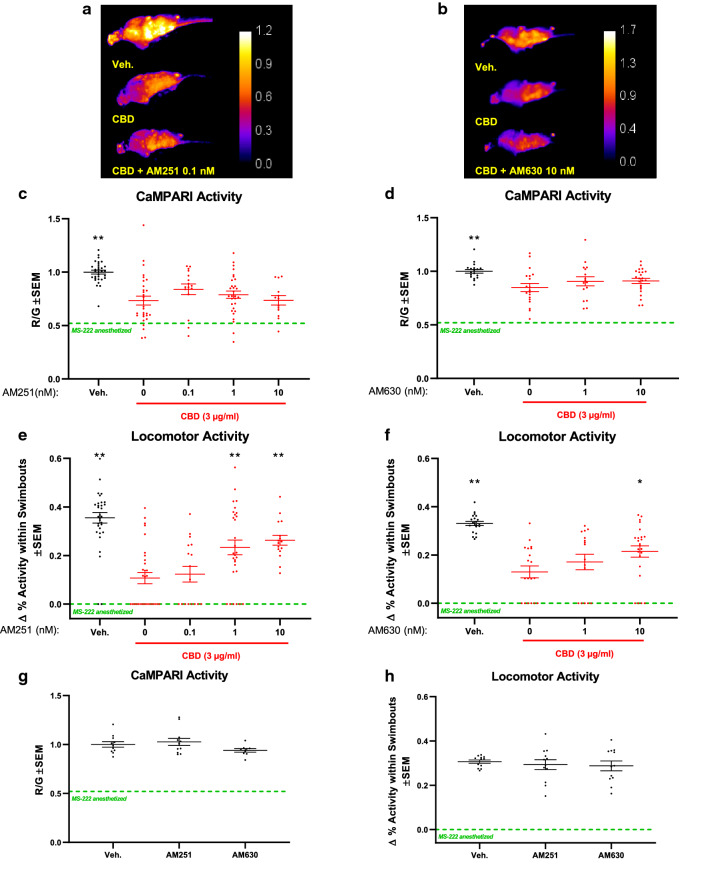
Cannabinoid receptor 1 (CB1R) and CB2R are both involved in CBD-mediated reduction of locomotor activity. Zebrafish larvae were exposed to CBD (3 µg/ml) with CB1R- and CB2R inhibitors, AM251 and AM630, respectively. (**a**) and (**b**), Exemplar CaMPARI heat maps showing R/G ratios obtained at 4 dpf. (**c**) and (**d**), Corresponding quantifications showing CBD mediated-reduction of neural activity is not fully rescued when CB1R is inhibited with AM251 or CB2R with AM630 in the nanomolar (nM) range. (**e**) and (**f**), Locomotor activity at 5 dpf shows a clear rescue when inhibiting either CB1R or CB2R with 0.1 nM AM251, or 10 nM AM630, respectively. (**g**) and (**h**), shows that applying AM251 or AM630 without CBD did not affect CaMPARI activity or locomotion. R/G is indicated by the calibration bar. All samples contained the same amount of vehicle DMSO (0.1%) and MeOH (0.3%) including the vehicle control (Veh.). Green-dashed lines depict mean values for MS-222 anesthetized samples. Biological replicates are n = 13–30. *is *p* < 0.05; **p < 0.01 compared to CBD.

Some reports suggest that CBD mediates its actions through CB1- and CB2-receptors^25^. To test whether these two receptors are involved in the neuronal effects mediated by CBD in vivo, we used the CB1R inverse agonist AM251 and the CB2R antagonist AM630. Previous findings support the specificity of CB1R and CB2R inhibitors in zebrafish^44,45^. For instance, a recent study showed that CB2Rs are required for the action of AM630 on zebrafish behaviour^44^. Importantly, the impact of AM630 on wildtype larvae was not detectable when applied to *cnr2*^−/−^ mutant larvae (CB2R knockouts), thereby supporting drug specificity. Moreover, AM630 treated zebrafish showed a very similar photo-dependent response compared to the CB2R knockout^44^. Similarly, either knocking out CB1R or inhibiting CB1R with AM251 caused a similar response in rescuing locomotor activity when the activity was suppressed with the cannabinoid-receptor agonist, WIN55,212-2^45^. In that study, the three metrics that were measured (acclimatization, dark-adaption, and recovery) for locomotion displayed arguably the same rescue-pattern in *cnr1*^−/−^ mutant and AM251 treated larvae^45^. In the present study, both inhibitors were used in the nanomolar range, which is considered modest and which ensures a level of specificity towards their respective receptors (AM251, IC50 = 8 nM towards CB1R; AM630, IC50 = 31.2 nM towards CB2R). As described previously, the experimental timeline is as depicted in Fig. 2b, with CBD being added immediately after AM251 and AM630. When analyzing CaMPARI activity, our results revealed that the CBD-mediated reduction in neural activity could not be completely prevented with concentrations from 0.1 to 10 nM AM251 or AM630 in the presence of 3 µg/ml of CBD (Fig. 4a–d). In contrast, the CBD-mediated reduction of locomotor activity was prevented with a concentration of AM251 as low as 1 nM (Fig. 4e) or with 10 nM of AM630 (Fig. 4f). Importantly, neither inhibitor had an effect on locomotion when used on their own, in the absence of CBD (Fig. 4g, h). This finding excludes the possibility that the effects of AM251 or AM630 on locomotion were independent of CBD and rather specific towards CB1R and CB2R, respectively. Taken together, these findings suggest a role for both CB1R and CB2R in the CBD-mediated effects on locomotor activity and that inhibiting CB1R and CB2R can ameliorate the negative impacts of early CBD on late CNS function, at least with respect to locomotion.

## Discussion

In some jurisdictions, including Canada, cannabis has been legalized despite the lack of mechanistic understanding and health implications. Cannabis consumption has increased in the past decade^46,47^ and recent reports associate prenatal cannabis consumption with stillbirths and the neurodevelopmental syndrome, autism spectrum disorder^48,49^. Yet, cannabis and CBD oil have been used to treat labor pain^50^. In animal models, such as rats and chicken, cannabis has been shown to have detrimental effects on developing embryos^10,51^. Recent evidence reveals that early exposure to CBD and THC impacts the overall development in zebrafish^13^. Interestingly, the effects of CBD and THC were delayed and were observed much later in development after the exposure had ended.

In vitro, CBD and THC can function through CB1R and CB2R, which are expressed in neuronal tissues^17–25^. A goal of this study was to investigate the effects of CBD and how it might interact with THC during early development to impact neural activity in older larvae. We implemented CaMPARI transgenic zebrafish as a read-out of neural activity and supplemented this approach by measuring the locomotor activity of the same larvae. CaMPARI has proven to be a relatively straightforward and economical neural integrator that can be used in a well-format with the zebrafish animal model. We found that neural activity was reduced in zebrafish larvae following brief exposure to THC and CBD early in development. Surprisingly, CBD had similar impacts on neural activity at lower concentrations compared to THC. Notably, the impacts of CBD were increased by sub-effective doses of THC. Our data quantifying locomotion suggested that these CBD-mediated effects involve CB1R and CB2R.

### CaMPARI assay

We optimized the workflow and photoconversion of CaMPARI such that this innovation can be scalable to a large number of compounds in future studies. Zebrafish larvae are often used in a 96-well plate format for quantifying locomotion^39,52–56^. Nevertheless, locomotor activity is not a direct measurement of neural activity. Calcium sensors, such as the GCaMP series^57,58^, the more recently introduced GECO^59,60^, and CaMPARI, offer opportunity for more direct measures of neural activity. Importantly, CaMPARI provides a temporal-snapshot of neural activity in the freely swimming animal, which can be anesthetized and imaged after the experiment^34^. Furthermore, the ratiometric red/green signals are imaged together and mitigate inter-individual differences arising from different expression levels or variances due to tissue depth. We optimized conditions for a well-plate set-up analyzing neural activity in the optic tectum and hindbrain. Future work might refine the method to quantify less defined brain areas or specific neurons.

In general, there seems to be a good correlation between neural activity measured by CaMPARI and locomotor activity^61,62^. However, some findings may be more sensitive towards one assay vs another, as described above. Furthermore, when interpreting our data, it is important to consider that when neural activity is very low, for instance when anesthetized, a clear red to green CaMPARI ratio can be still measured. This is unlikely due to autofluoresence because larvae that were not photoconverted yielded zero CaMPARI activity. We cannot exclude the possibility that due to longer photoconversion times some CaMPARI was converted into the red fluorescence version even when anesthetized. It is well established that when anesthetized with MS-222, neurons are barely active^63,64^. Indeed, the behavioural assay shows no locomotion in the presence of MS-222 and we used this assay as a supplement in this study. Thus, we believe this method is a suitable technique to measure substantial changes in neural activity in the larger parts of the zebrafish CNS. For future studies in connection to epilepsy this innovation may be particularly helpful as some studies use convulstants, PTZ or 4-AP, to simulate seizures^62,65^. Indeed, we found that both, PTZ and 4-AP, induce CaMPARI activity. Altogether, the advantages with this method outweigh its limitations and could serve for future studies, especially in connection with epilepsy.

Several competing technologies were considered prior to designing this study, in the hope that we could build upon and complement these potent approaches. Other than behavioural assays described above, the GCaMP series has been of interest^57,58^. However, the larvae have to be analyzed with high-resolution live microscopy during the experiment and movement of the larvae would add considerable variability. The assays would be data-intensive and technically challenging, particularly where large sample sizes and different experimental groups are difficult to obtain (especially in vivo). Additionally, we were interested in measuring outputs from freely behaving animals because constraining them for imaging would likely impact the results and would be time-consuming. Other widely used markers for neural activity include the immediate-early gene *c-fos* and phosphorylation of ERK^65,66^. However, CaMPARI is more temporally specific due to its immediate photoconversion. In addition, both markers (*c-fos* and ERK) are not only less tissue specific, but also respond to stress and other factors. The *elavl3* promoter that drives CaMPARI ensures CNS-specific expression. Overall, CaMPARI is a practical direct output of neural activity and thus the chosen innovation for this study.

### Delayed implications after early embryonic exposure of CBD and THC

CBD and THC have both been implicated in negatively impacting embryonic neurodevelopment. Later abnormalities following acute early exposure to these cannabinoids have only recently been investigated^13^. Here, early exposure of CBD and THC during the first 10 h of embryonic development reduced neural activity in a dose-dependent manner when measured 4 days later. These later effects may be due to abnormal neurodevelopment and/or the slow release of the cannabinoids from the tissue due to their lipophilic nature. Consistent with this finding, zebrafish larvae treated with a similar exposure (5–10 hpf) to cannabinoids have shown effects that may be due abnormal development (at 5 dpf). Those effects were not exclusive to neurons, but also included morphological abnormalities, decreased survival rate, decreased mEPCs activity (may relate to muscle development), decreased heart rate and delayed hatching^13^. Considering the specific time-window of exposure (first 10 h in development), an abrupt effect on the developmental program including neurons seems plausible. Interestingly, CBD seems to impact neural activity at a lower concentrations than THC, as observed herein and during our previous study^13^. It is important to acknowledge that CBD consumption has been documented to help with seizures and cancer by reducing pain^67,68^. Nevertheless, our study suggests caution regarding its use during pregnancies.

This study reveals some complexity when assessing the impacts of CBD and THC together. Their combined effects were more potent in decreasing later neural activity, locomotion and immunolabelling of reticulospinal neurons, suggesting there might be an impact on neuronal integrity^42,69^. Overall, these findings were surprising because CBD has been found to negate the effects of THC on CB1R^27^. One potential explanation is that at higher concentrations both CBD and THC alter the developmental program, which could underlie the abnormal morphologies reported previously^13^. In this study, we observed similar morphologies, which may have affected our results. However, because there were decreases in neural activity and locomotion at concentrations that did not show major morphological changes^13^, it is unlikely that the effects of CBD and THC are due to abnormal morphologies alone. Moreover, it is possible that the impact of higher concentrations of CBD and THC on developing brains is different compared to lower concentrations on fully developed brains. Finally, CBD and THC serve as ligands for several types of receptor with different affinities^25^, and another possibility is that at high concentrations, CBD and THC affect receptors other than CB1R and CB2R.

Aside from the impact of CBD and THC together, an additional interesting finding is that the most prominent action of early exposure to CBD is on subsequent locomotion. It remains unclear if that is due to CBD impacting muscle or other developmental aspects. It is also likely that the intertwined effects of CBD and THC are dose-dependent and past observations from higher CBD and THC doses suggest that the development of additional morphological deficits may be important for future considerations.

### Potential mechanism involved in the delayed effect of CBD and THC

At this stage, it is unclear what mechanisms account for the reduction in neural activity later in development when embryos are exposed briefly to CBD and THC in the first hours after fertilization. We were especially interested in the less widely studied CBD. The potential mechanisms of acute cannabinoid exposure on later neuromuscular outputs are diverse. Using HEK cells several receptor candidates have been identified that were shown to interact with CBD^25^. A recent study supports a functional role for the actions of CBD through CB1R and CB2R in zebrafish^32^. Herein, CB1R and CB2R were found to be required for the impact of CBD on locomotor activity. Nanomolar concentrations of receptor inhibitors partially rescued CBD effects. Previous studies have implied that CBD may bind to both CB1R and CB2R in vitro^25^ and the data in this study support these interactions in vivo. CBD has also been shown to cause abnormal craniofacial and brain development in mice and zebrafish, which seems to be mediated through CB1R and the hedgehog signaling pathway^16^. CB1R and CB2R are expressed very early in development on stem cell progenitors^70^. Therefore, developmental programs may be impacted such that changes persist until later, even when cannabinoids are no longer present in the circulation. Such changes may not be specific to the CNS as CBRs are not only expressed in neuronal progenitor cells, but also on stem cells forming other types of tissues^70^. This could explain our previous findings where CBD affected heart-rate and overall morphology after 5 dpf when exposed only briefly during gastrulation^11^. This study shows that CB1R and CB2R inhibition had little detectable effect on CBD impacting later neural activity. This might be an artifact of our CaMPARI method measuring general CNS activity in large areas, rather than resolving motor axons in detail, which have been shown to be affected previously^13^. We also acknowledge that pharmacological inhibitors may not completely block the CB1Rs and CB2Rs, or that they may have off-target effects. Finally, it is also possible that other CBD receptors such as GPR55 and TRPV1 are involved^26^, but this needs further investigation.

## Conclusion

In conclusion, both CBD and THC exposure during early embryonic stages had a negative impact on later neural activity, which was synergistic when the cannabinoids were combined. This suggests that the impact of cannabis on early development is likely greater than when using isolated compounds. Our findings also support an in vivo mechanism of CBD functioning through CB1R and CB2Rs. This study opens up a new path for investigating detailed mechanisms involving these and other receptors, especially in neurons that are affected by cannabinoid exposure early in development. Considerable evidence is accumulating that warns prenatal cannabis consumption can have negative consequences on neurodevelopmental disorders^3^ and further studies are warranted to fully understand the impacts on human development.

## Methods

### Animal ethics and exposure to drugs

Zebrafish maintenance was approved by the Animal Care & Use Committee: Biosciences at the University of Alberta and operated under the guidelines of the Canadian Council of Animal Care. The fish were maintained in the University of Alberta fish facility at 28 °C under a 14/10 light/dark cycle as previously described^71^ and all authors complied with ARRIVE (Animal Research: Reporting of In Vivo Experiments) guidelines^72^.

CaMPARI transgenic zebrafish were outcrossed with *Casper* strains not carrying the transgene. For the experimental setup, embryos were randomly collected and placed in egg water (60 µg/ml Instant Ocean) in groups of 20 embryos as early as practically possible within 0.5 hpf. Stock solutions of CBD and THC were obtained from Sigma at 1 mg/ml dissolved in methanol. Receptor inhibitors AM251 (Selleck Chemicals, Houston, TX, USA) and AM630 (Adooq Bioscience, Irvine, CA, USA) were dissolved in DMSO. Accordingly, zebrafish embryos were treated with vehicles or compounds as close to 1 hpf as practically possible and removed towards the end of gastrulation (10 hpf). Within experiments all treatments contained the same amount of methanol or DMSO, specific percentages are indicated in the figure legends. Compounds were removed with several washes and egg water was replaced every day until CaMPARI analysis. Not all larvae carried the transgene and were screened for green fluorescence. An increase in mortality and abnormal morphology was observed when treated with higher doses of CBD or THC, as reported previously^13^.

### Engineering CaMPARI transgenic zebrafish for integrative calcium imaging

Zebrafish expressing CaMPARI, i.e. the Tg[*elavl3*:CaMPARI (W391F + V398L)]^ua3144^ line were generated as described previously^61,62^. We re-derived CaMPARI fish in response to a federal moratorium limiting zebrafish import into Canada^73^.

### CaMPARI photoconversion

For photoconversion, bright green Tg[*elavl3*:CaMPARI (W391F + V398L)]^ua3144^ larvae at 4 dpf were placed in a 96-well plate containing 150 µl egg water (made as previously decribed^71^) and acclimatized for 2 h. The central 48 wells of the plate were exposed to 405 nm LED array (Loctite) for 300 s at a distance of 10 cm. LED array illuminated the plates entirely and evenly. The plates were floating in water at room temperature to ensure that the larvae did not overheat. For imaging, the larvae were anesthetized in 0.24 mg/mL tricaine methanesulfonate (MS-222, Sigma) following photoconversion. MS-222 was washed out and zebrafish were rested for a day prior to analysis of locomotor activity. Previously, CaMPARI was not optimized for well-plate-formats because a 10 s photoconversion did not yield sufficient red fluorescing CaMPARI to be detected reliably. The method used in this study does not require a multi-photon confocal microscope where the larvae need to be carefully embedded in agarose and placed against the microscope slide. Instead, the larvae can be imaged directly from the well after MS-222 treatment. This assay also complements more straightforward and more economical fluorescing microscopes for labs with a lower dedicated budget.

### CaMPARI analysis

The ratio of red to green fluorescent emissions (red photoconverted CaMPARI in ratio to green CAMPARI) was interpreted as relative neural activity, as previously defined^34^. Images were objectively obtained with an InCell 2000 microscope (GE Healthcare, US). 96-well plates were run using FITC (1 s exposure) and dsRed (2 s exposure) channels. Blinded experimentalists used ImageJ to quantify the fluorescence mean of the red (dsRed) and green (FITC) channels. The area of the optic and hindbrain area were quantified as those regions were most consistent in the view. A mask was applied to exclude noise outside of the CNS.

### Quantifying locomotor activity

The same zebrafish larvae being placed in the 96-well plate the day prior for CaMPARI analysis were objectively quantified for locomotion using Basler GenICaM (Basler acA 1300-60) scanning camera (75-mm f2.8 C-mount lens) and EthoVision XT-11.5 software by Noldus (Wageningen, Netherlands) as described previously^74^. Briefly, larvae were acclimatized for 3 h and one hour of movement was recorded from each individual larvae in each well. Beneath the plate an infrared backlight source was located and the scanning camera above. Activity was defined here as % pixel change within well between frames (recordings were at 25 frames per second) and quantified with EthoVision XT-11.5 software. This set-up allowed to see whether there is a correlation between level of neural activity and the the mean activity % within a swimbout (swimbout size). Swimbout size was shown to increase in response to the convulsant PTZ previously^52^ and here it can be a useful marker for neural activity. Transparent *Casper* strain zebrafish^75^ were unable to be tracked as ususal with Noldus. Therefore, quantification of swimbouts provided an additional advantage. Clear movement was detected above the noise treshold due to movement of the pigmented eyes. Although the % swimbout is small, reliable measurements were obtained. Only clear swimbouts were quantified, which were set above a treshold of 0.1% of activity. Figure 1f shows that measuring the mean activity of a swimbout is a useful tool in obtaining output that is clearly diminished in presence of MS-222. In addition, the output also shows sensitivity in freely swimming larvae.

### RMO44 staining

The zebrafish used in this experiment were of the Tuebingen Longfin strain. Set-up was similar as described above. Following the experiment, larvae were stained with anti-RMO44 (Thermo Fisher Scientific, Waltham, MA, USA) and imaged using a Zeiss LSM 710 confocal microscope (Oberkochen, Germany) as described elsewhere^76^.

### Statistics

GraphPad Prism Software (Version 7, GraphPad, San Diego, CA) was used to analyze statistics from data obtained from our CaMPARI assay or tracking locomotor activity. All the data were presented as mean ± SEM (standard error of the mean). Statistical significance of *p* < 0.05 was determined using a non-parametric t-test between two groups followed by a Mann–Whitney analysis, if appropriate. Multiple groups were compared using One-Way ANOVA with Dunnett's multiple comparisons test. Pearson *r* from correlation analysis was also determined where indicated.

## Supplementary Information

Supplementary Information 1.

Supplementary Information 2.
