# Supplementary material for: Medium-throughput zebrafish optogenetic platform identifies deficits in subsequent neural activity following brief early exposure to cannabidiol and Δ9-tetrahydrocannabinol
Source: Sci Rep. 2021 Jun 1;11:11515. doi: 10.1038/s41598-021-90902-3 (PMC8169761; doi:10.1038/s41598-021-90902-3)
Supplement: Supplementary file 1 — Supplementary Information 1. [file 41598_2021_90902_MOESM1_ESM.docx]

**Figure S1: Supplementary figure accompanying Fig.2.** Re-plotting data to illustrate that CBD shows an effect at lower concentration than THC. Quantifications of CaMPARI activity (left) or locomotor activity (right) showing a statistical difference between CBD and THC at 4 or 5 days post-fertilization when adding 3.0 µg/ml of either cannabinoids. Green-dashed lines depict mean values for MS-222 anesthetized samples. Biological replicates are n=14-28. Statistics using unpaired t-test shows ** p<0.01 compared to CBD.

**Figure S2: Supplementary figure accompanying Fig.2.** Re-plotting data from groups that were treated with vehicle, CBD (3 µg/ml) or THC (6 µg/ml) to assess correlation between CaMPARI activity vs. Locomotor activity within the same larvae. There is a significant positive linear correlation between neural activity and locomotor activity when corresponding vehicle controls (black circles) were plotted with either CBD (**a**; red circles) or THC (b; blue circles). Green-dashed lines depict mean values for MS-222 anesthetized samples.

**Figure S3: Supplementary figure accompanying Fig.3.** Additive effects in reducing neural activity are also obtained at lower CBD concentrations while THC concentration is kept constant at 2 µg/ml. (**a**) Exemplar CaMPARI activity heat maps obtained at 4 days post-fertilization show an effect at 0.5 and 1.5 µg/ml of CBD when combined with 2 µg/ml THC (blue) compared to CBD alone (red), as illustrated by quantifications in (**b**). (**c**) Locomotor activity from the same well at 5 dpf. R/G as indicated by the calibration bar. Green-dashed lines depict mean values for MS-222 anesthetized samples. Biological replicates are n=14-28. * is compared to CBD; ^#^ is compared to Vehicle. One symbol is p<0.05; two symbols are p<0.01.

**Figure S4: Supplementary figure accompanying Fig.3.** RMO44 staining of reticulospinal neurons in the hindbrain shows a decrease in fluorescence intensities when combining CBD and THC at 2 µg/ml at 5 dpf compared to when CBD or THC was added alone. Images show biological replicates (n=3), left to right, from corresponding treatment group, top to bottom.
