# Supplementary material for: Medium-throughput zebrafish optogenetic platform identifies deficits in subsequent neural activity following brief early exposure to cannabidiol and Δ9-tetrahydrocannabinol
Source: Sci Rep. 2021 Jun 1;11:11515. doi: 10.1038/s41598-021-90902-3 (PMC8169761; doi:10.1038/s41598-021-90902-3)

Supplementary Information

Medium-throughput zebrafish optogenetic platform identifies deficits in subsequent neural activity following brief early exposure to cannabidiol and Δ^9-tetrahydrocannabinol

Richard Kanyo[1,2], Md Ruhul Amin[2], Laszlo F. Locskai[1,2], Danika D. Bouvier [2], Alexandria M. Olthuis[2], W. Ted Allison[1,2,3], and Declan W. Ali [2, *]

[1] Centre for Prions & Protein Folding Disease, University of Alberta, Edmonton AB, T6G 2M8

[2] Department of Biological Sciences, University of Alberta, Edmonton AB, T6G 2E9

[3] Department of Medical Genetics, University of Alberta, Edmonton AB, T6G 2H7, Canada

* Author for correspondence (phone: (780) 492-6094; fax: (780) 492-9234; email: declan.ali@ualberta.ca)

Fig. S1

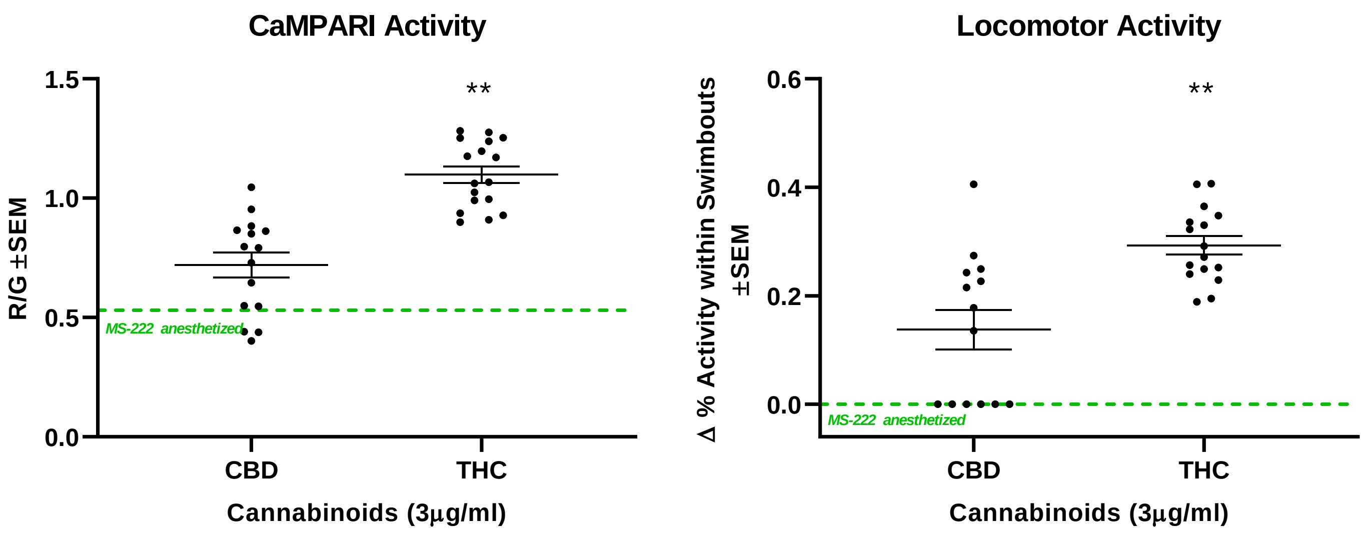
CaMPARI Activity
R/G ±SEM
1.5
1.0
0.5
0.0
**
MS-222 anesthetized
CBD
THC
Cannabinoids (3µg/ml)
Locomotor Activity
Δ % Activity within Swimbouts ±SEM
0.6
0.4
0.2
0.0
**
MS-222 anesthetized
CBD
THC
Cannabinoids (3µg/ml)

Fig. S2

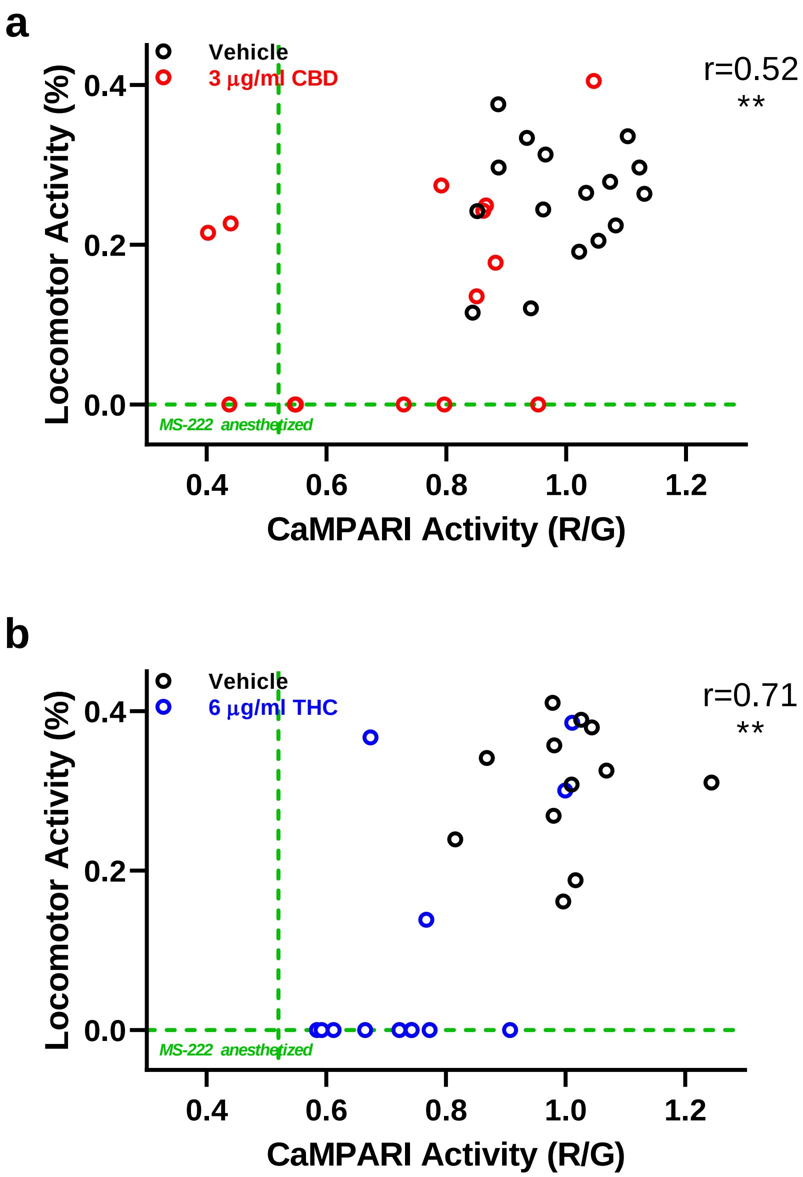
a
Vehicle
3 µg/ml CBD
r=0.52
**
Locomotor Activity (%)
0.4
0.2
0.0
MS-222 anesthetized
0.4
0.6
0.8
1.0
1.2
CaMPARI Activity (R/G)
b
Vehicle
6 µg/ml THC
r=0.71
**
Locomotor Activity (%)
0.4
0.2
0.0
MS-222 anesthetized
0.4
0.6
0.8
1.0
1.2
CaMPARI Activity (R/G)

Fig. S3

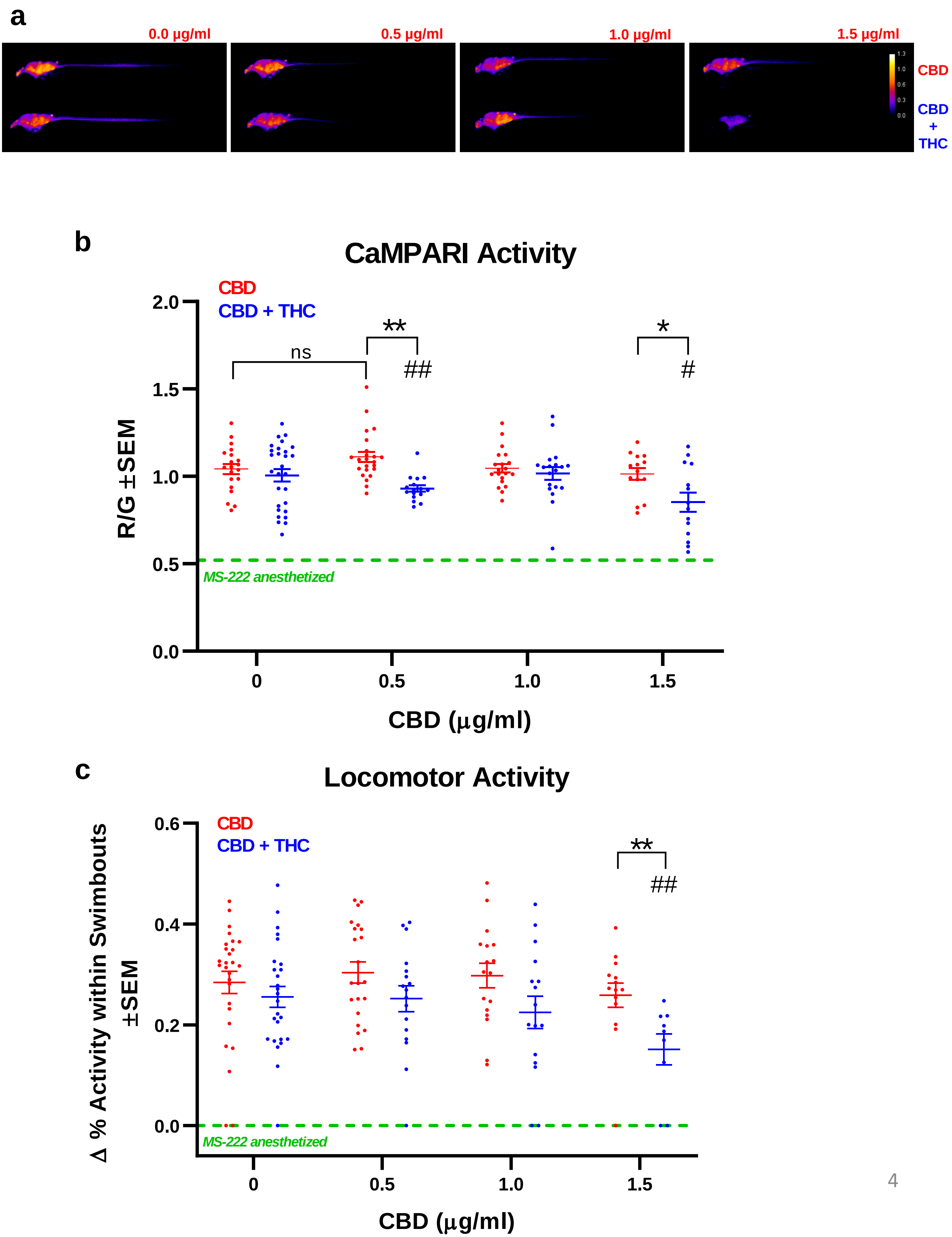
a
0.0 µg/ml
0.5 µg/ml
1.0 µg/ml
1.5 µg/ml
1.3
1.0
0.6
0.3
0.0
CBD
CBD + THC
b
CaMPARI Activity
CBD
CBD + THC
ns
**
##
*
#
MS-222 anesthetized
R/G ±SEM
2.0
1.5
1.0
0.5
0.0
0
0.5
1.0
1.5
CBD (µg/ml)
c
Locomotor Activity
CBD
CBD + THC
**
##
MS-222 anesthetized
Δ % Activity within Swimbouts ±SEM
0.6
0.4
0.2
0.0
0
0.5
1.0
1.5
CBD (µg/ml)

Fig. S4

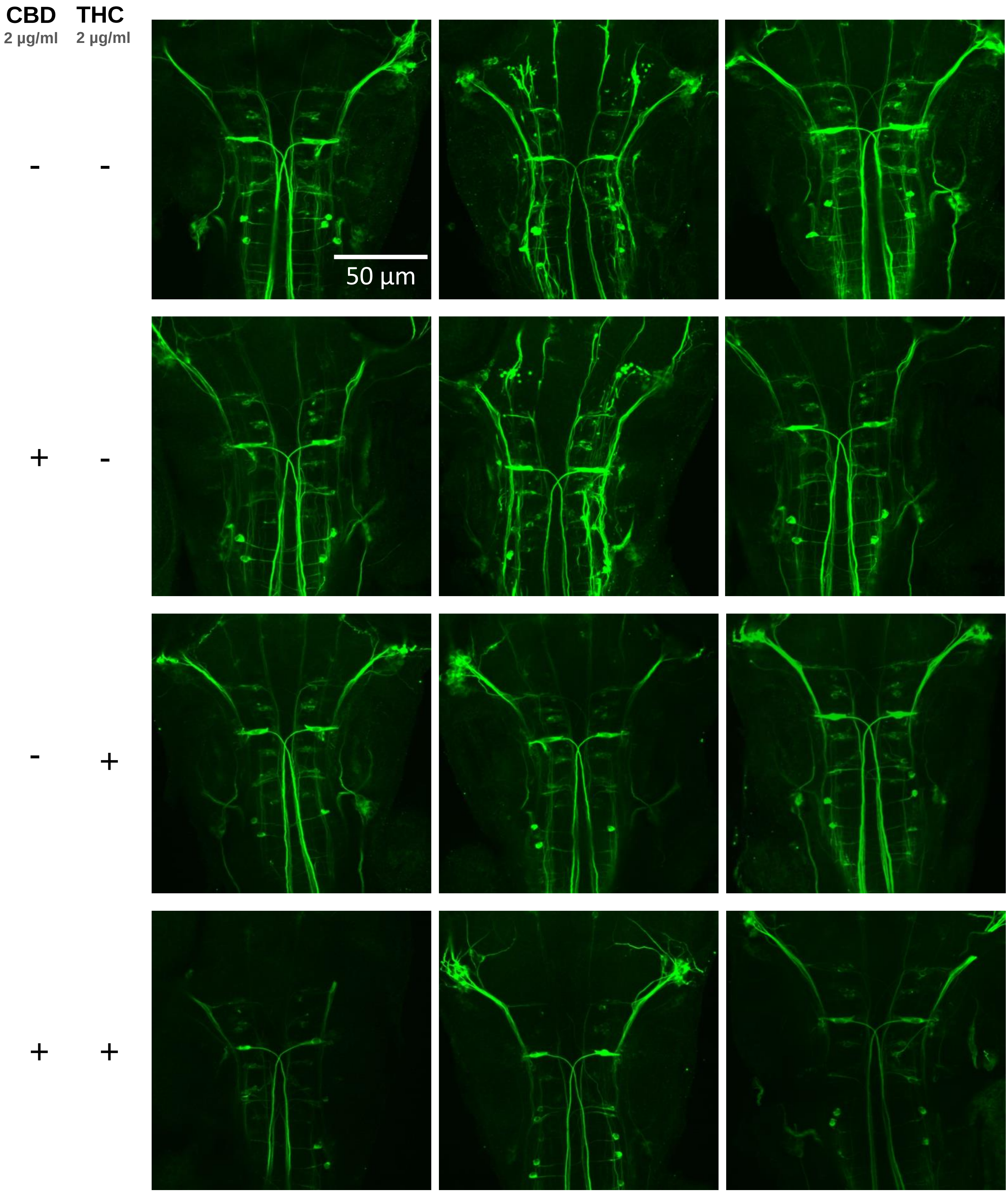

Supplement: Supplementary file 2 — Supplementary Information 2. [file 41598_2021_90902_MOESM2_ESM.pdf]
